# Vaccination Offer during the Occupational Health Surveillance Program for Healthcare Workers and Suitability to Work: An Italian Retrospective Cohort Study

**DOI:** 10.3390/vaccines10101633

**Published:** 2022-09-28

**Authors:** Francesco Paolo Bianchi, Pasquale Stefanizzi, Luigi De Maria, Andrea Martinelli, Giusy Diella, Angela Maria Vittoria Larocca, Luigi Vimercati, Silvio Tafuri

**Affiliations:** 1 Interdisciplinary Department of Medicine, Aldo Moro University of Bari, 70124 Bari, Italy; 2 Hygiene Unit, Bari Policlinico General Hospital, 70124 Bari, Italy

**Keywords:** healthcare workers vaccination, biological risk assessment, vaccination hesitancy, occupational medical examination

## Abstract

The active immunization of health care workers (HCWs) is a crucial measure to avoid nosocomial infection; nevertheless, vaccine coverage (VC) among health personnel in Italy is unsatisfactory. To improve VC in the healthcare set, the Hygiene and Occupational Medicine departments of Bari Policlinico General University Hospital applied a specific program. The operative procedure demands that in the context of the occupational medical examination, all workers are evaluated for susceptibility to vaccine-preventable diseases (VDPs), with immunization prophylaxis offered to those determined to be susceptible. This study analyzed data from workers who attended the biological risk assessment protocol from December 2017 to October 2021 (*n* = 1477), who were evaluated for the immune status for measles, mumps, rubella, and varicella. Among the enrolled subjects, non-protective antibody titers were higher for measles and mumps (13%), followed by rubella (11%) and varicella (8%). Appropriate vaccinations were offered to all susceptible HCWs, and HCWs were re-tested one month after immunization. The seroconversion rate after the administration of one or more booster dose(s) was over 80%. Overall, 2.5% of the subjects refused the offered vaccine(s); the main determinant of immunization compliance was younger age (aOR = 0.86; 95%CI = 0.80–0.92). Especially during the COVID-19 pandemic, VPDs may still present a hazard in nosocomial environment. Our experience suggests that, despite hospital procedures and dedicated human assets, satisfactory VC cannot be reached without the provision of federal regulations. Nevertheless, public health policymakers have to improve the promotion of vaccine prophylaxis and education to reach higher VC.

## 1. Introduction

Healthcare workers (HCWs) are exposed to various workplace risks, specifically biological infectious diseases [1]; furthermore, the spread of pathogens to coworkers and patients is possible. Vaccination of health personnel is a prevention policy that can control the risk of spread of vaccine-preventable diseases (VDP), particularly to the most frail subjects (e.g., oncological or immunocompromised patients) [2]. Furthermore, it is also recommended in order to assure healthcare delivery during outbreaks [3] and reduces staff absenteeism [4].

The most updated guidelines strongly recommend that health personnel should be evaluated for immune status regarding measles, mumps, rubella, and varicella vaccines and that those susceptible receive the appropriate vaccine [2,5]. Considering that in Italy (and most European nations) immunization prophylaxis is just recommended [3,6,7,8,9], some clusters of measles or varicella have been reported in nosocomial environments [10,11,12], with many cases among health personnel [13,14,15]. In Italy, vaccination coverage (VC) values among health personnel are not systematically recorded [7]. Anyway, the studies available in scientific literature have reported low VC among Italian health staff [3,16,17,18,19]. Therefore, immunization of Italian health personnel is an undercurrent public health topic; indeed, the National Vaccine Advisory Committee has settled various strategies to improve influenza immunization rates in healthcare environment [20], but no standardized protocols for other recommended vaccinations for health personnel [2,5,21]. In a 2013 study [22], education courses to increase the understanding and tolerance of vaccination among HCWs were proposed; the authors also suggested a major role of Public Health doctors in offering vaccination-related information to health staff in the context of medical examinations [23].

In order to increase immunization adherence among health personnel and rise VC in healthcare environment, the Hygiene and Occupational Medicine departments of Bari Policlinico General University Hospital (~1000-bed hospital) designed and applied a vaccination protocol that requires all employees to be evaluated for immunity/susceptibility to VPDs (hepatitis B, measles, mumps, rubella, varicella, tetanus, meningococcus) during the occupational medical examination, offering vaccination to those found to be susceptible.

This analysis was conducted in Puglia (southern Italy, almost 4,000,000 inhabitants) and its aim was to evaluate the susceptibility to measles, mumps, rubella, and varicella of a sample of health personnel working at the Bari Policlinico and the attitude to immunization. Explanations for immunization refusal were also assessed to develop strategies to increase vaccine compliance among health personnel.

## 2. Materials and Methods

The paper model is a retrospective cohort study.

In November 2017, the departments of Hygiene and Occupational Medicine of the Bari Policlinico have drawn up the “Operative protocol for the vaccination prophylaxis of healthcare workers” [24]. The procedure fulfilled the national guidelines [5,21] regarding vaccine prophylaxis in health personnel; it comprised a biohazard prevention protocol for health staff during their pre-recruitment medical examination or the routine scheduled occupational medical check [21]. This protocol has been implemented since December 2017 as a pilot procedure in Italy: the department of Occupational Medicine calendars the occupational medical examinations for health staff; a blood sample was taken and then examined using chemiluminescence techniques. Blood test results disclose whether the subject is seroprotected against measles (IgG titer > 16.5 Arbitrary Unit [AU]/mL), mumps (IgG titer > 11 AU/mL), rubella (IgG titer > 165 International Unit [IU]/mL), and varicella (IgG titer > 165 mIU/mL), among other VPDs; a threshold was defined according to the recommendation of blood test kits. The immunization status of enrolled subjects was assessed using the Regional Immunization Database (GIAVA) [25]. Susceptible subjects were then invited to the Hygiene department to define the appropriate vaccine prophylaxis, if needed. The vaccine advising was executed by public health doctors who were experts in vaccinology.

For HCWs with a non-protective IgG titer, and/or not vaccinated for MMR/Vzv, appropriate vaccination (2 doses at 0–1 months) was offered. For seronegative subjects who received ≥ 1 dose(s) of vaccine a booster dose was administered, and a blood sample was acquired after 28 days to re-test IgG titer; if it exceeds the cut-off, the subject is categorized as seroconverted, and no additional doses of vaccine are required. If the IgG titer is negative, another shot of vaccine is administered (28 days after the first booster shot); after 28 days the IgG titers were re-assessed. Seronegative subjects deprived of an accessible vaccination schedule were considered never immunized. Live attenuated vaccines (M-M-RVAXPRO/VALRILIX) were administered subcutaneously into the deltoid.

Immunization prophylaxis is not mandatory and health staff may reject it. Informed consent was systematically collected. All immunized subjects underwent a 1 month follow-up to evaluate the insurgence of adverse events, and they were instructed to contact the Hygiene department in case of adverse events.

Upon completion of the procedure, the Hygiene department shows a report to the Occupational Medicine department on the subjects’ immunological status and any measure implemented. Lastly, the Occupational Health doctor expresses a judgment listing placement alternative for each employee considering the susceptibility status and risk assessment. For non-immunized subjects who reject vaccination prophylaxis, exclusion from healthcare environments with patients at high infectious risk is recommended; a physician who is not immunized against rubella is not fit for a job in the Obstetrics ward, due to the extremely susceptible patients.

Our sample included health personnel who participated in the biological risk assessment protocol (December 2017–October 2021). The following information were obtained: age, sex, medical specialty, professional category, history of chronic diseases (yes/no) available vaccination schedule (yes/no), MMR/Vzv IgG titers at baseline, vaccinations administered (yes/no), IgG titer after booster shot(s), and vaccination refusal (yes/no).

Data were entered into an Excel worksheet and analyzed by STATA MP17 software. Continuous variables were described as mean ± standard deviation and range, and categorical variables as proportions, with a 95% confidence interval (95%CI) when appropriate. Antibody titer was expressed as geometric mean titer (GMT).

Multivariate logistic regression models were performed to assess

- determinants of vaccination refusal
- determinants of susceptibility to serological evaluation.

In both cases, determinants were age (years), sex, immunization status, chronic diseases (yes/no), allergies (yes/no), medical specialty, and professional category. The adjusted Odds Ratio (aOR) was calculated along with the 95%CI. Pearson or Hosmer–Lemeshow’s chi-squared tests were used to evaluate the goodness-of-fit of multivariate logistic regression models [26]. Multicollinearity between determinants were tested, without evidence of it.

A *p*-value < 0.05 was considered statistically significant for all tests.

## 3. Results

From December 2017 to October 2021, 1477 HCWs were tested; the characteristic of the sample is reported in Table 1.

Immunization status by vaccine type is described in Table 2.

**Figure 1:**
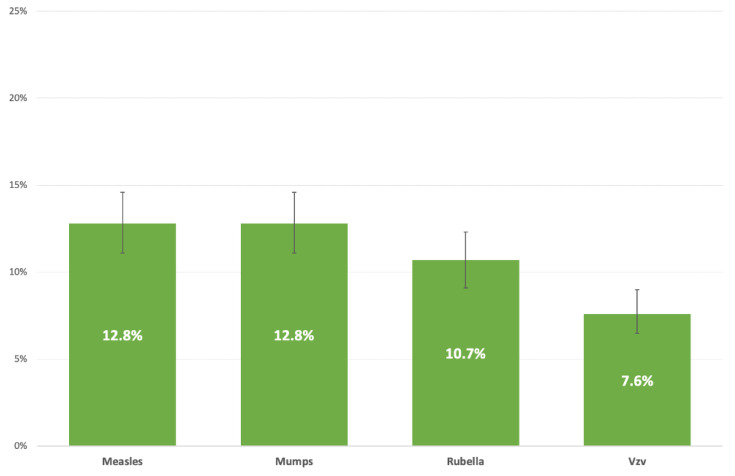
Proportion (%) of healthcare workers without circulating antibodies to measles, mumps, rubella, and varicella, per disease.

Overall, 1473/1477 (99.7%) subjects were examined for anti-measles and anti-rubella IgG, 1471/1477 (99.6%) for anti-mumps IgG, and 1470/1477 (99.5%) for anti-Vzv IgG. The proportion of subjects who had no circulating antibodies to each infection is reported in Figure 1. Results of multivariate analysis of determinants of serosusceptibility at enrollment are shown in Table 3.

The geometric mean titers (GMTs) for the infection investigated in immune subjects were anti-measles IgG 190.0 (95%CI = 182.6–216.8), anti-mumps IgG 94.3 (95%CI = 87.7–101.4), anti-rubella IgG 41.4 (95%CI = 37.9–45.3), and anti-Vzv IgG 857.6 (95%CI = 808.8–909.3).

The health personnel offered vaccination prophylaxis, per vaccine, and the seroconversion proportion in re-evaluated HCWs are reported in Table 4.

Overall, 516/1477(34.9%) subjects needed vaccine prophylaxis; it was offered to 364/516 (70.5%) of them. Particularly, the MMR vaccine was offered to 309/533 (58.0%) susceptibles for MMR, and the anti-Vzv vaccine was offered to 82/111 (73.8%) susceptibles (many subjects were susceptible to more than one infection). Overall, 13/516 (2.5%) subjects refused at least one vaccine, specifically, 8/309 (2.5%) of measles or mumps or rubella susceptibles refused the MMR vaccine, and 5/82 (6.1%) of varicella susceptibles refused the anti-Vzv vaccine. The multivariate regression showed a statistically significant association between vaccination refusal and older age (aOR = 1.16; 95%CI = 1.09–1.25); no other factor was related to vaccination refusal (*p* > 0.05; Table 5).

No serious and/or long-term adverse reactions during the 1 month follow-up were reported. The most frequently described events were pain at the injection spot, unimportant fever, and, infrequently, lymphadenopathy. All of these reactions retrogressed over the next few days without consequences.

At the end of the study period, 3.5% of tested subjects were still not sero-protected for measles or had not received vaccine prophylaxis, 5.2% for mumps, 4.3% for rubella, and 2.6% for Vzv.

## 4. Discussion

Our study reported a very low VC for anti-MMR and anti-Vzv; indeed, anti-MMR/Vzv vaccines have been endorsed since the 1980s–1990s, but they have only been made mandatory for children since 2017. Therefore, pediatric immunization was not required for health personnel in our paper. Indeed, most of them contracted measles and varicella during their lifetime because these infections were endemic in Apulia until 2006.

Regarding circulating antibodies, susceptibility was higher for measles and mumps (12.8%), followed by rubella (10.7%) and varicella (7.6%). The MMR susceptibility in our sample was comparable to that reported in the scientific literature. In a 2020 meta-analysis, 9.1% of Italian HCWs were seronegative for measles [27]. The percentage of HCWs negative for anti-measles IgG was slightly lower than that determined in two studies on students and residents of Medical School conducted by our research team [28,29], with 15% of susceptibles. A 2019 paper [30] showed an overall seropositivity of measles on 7411 South Korean HCWs born from 1952 to 1995; this value declined from 85% in the 1986 birth cohort to 42% in the 1995 birth cohort. Regarding mumps, a 2013 study from Spain [31] assessed that 13% of 639 HCWs were susceptible to mumps, and a 2014 paper [32] reported a serosusceptibility of 11% in Japanese health personnel aged ≤ 29 years. On the other hand, a 2020 Italian study [33] of 2000 fully vaccinated medical students showed a prevalence of susceptibility equal to 6%. In the same sample, for 181 (9%) medical students and residents, IgG against rubella was undetectable [34]; a Japanese study [32] reported that 11% of 1811 tested HCWs were serosusceptible. The susceptibility of health personnel to Vzv appears to be country-dependent, ranging from 5% to 50% [32,35]. Two Italian studies [36,37] evaluated circulating anti-Vzv IgG in fully vaccinated young HCWs and reported a prevalence of serosusceptibility ranging from 21 to 34%. This strong dissimilarity could be associated with the dissimilarity in the average age and immunization status of the populations being analyzed.

Older age is associated with a higher probability of detection of circulating antibodies; in fact, our study showed a greater risk of sero-susceptibility in subjects born in the post-vaccination era. Therefore, these subjects probably did not come into contact with the wild virus, whose circulation had decreased since the start of immunization campaigns. Although vaccine-induced immune response is qualitatively comparable to that induced by infection, antibody titers are usually inferior after vaccination [38]. Considering mumps, multivariate analysis evidenced that male subjects were less prone than females to have circulating anti-mumps IgG (aOR = 1.43; 95%CI = 1.01–2.03). Sex differences in response to immunization or infection have been studied in several papers [39], with females commonly having more successful immune responses, with immunological, genetic, hormonal, environmental, and microbiotic factors contributing to this distinction. Finally, the role of vaccination as a determinant of serosusceptibility is different for rubella and varicella. MMR vaccination seems to be a protective factor for seroprotection, probably because of the goal of rubella (and measles) elimination and the associated vaccination campaign, whereas the Vzv-vaccine seems to be correlated with an improved risk of susceptibility at serologic evaluation. Indeed, as reported by many studies in the literature [36,37], the time from Vzv vaccination to evaluation of antibody level is a major factor of the decay in serum of circulating antibodies and thus protection against the wild virus; in particular, it appears that for the MMR vaccine, the duration of circulating antibodies is more than twice that of Vzv vaccine [36].

For all vaccines, the seroconversion proportion after booster vaccine shots was high (over 80%), confirming what has already been evidenced in the literature [24,28,29,33,34].

Overall, 2.5% of health personnel rejected the proposed vaccine(s), with anti-Vzv refused more often (6%) compared with the MMR vaccine (2.5%); the principal determinant of a good attitude around vaccination was younger age (*p* < 0.001). Previous studies have shown that older healthcare providers are unwilling to get an influenza vaccine, while younger HCWs are more acquiescent [16,40,41,42]. One of the most frequent reasons for vaccination hesitancy was the absence of active offerings. The above-described operating procedure is one method for dealing with this concern. Squeri et al., in a 2017 study [4], showed that a higher number of years of job service was a factor of bad vaccination attitude, in concordance with our data. Another factor of bad vaccination attitude was the low awareness of risk related to VPDs [43], which may explain the denial to be vaccinated against infections seen as uncommon or not threatening.

The safety of booster vaccination reported by our results is consistent with evidence in the literature [44].

A fact of strength of our paper was the considerable sample size (1477 HCWs). Moreover, a previous study of our research team assessed the biological risks of health personnel in the set of an Occupational Medical examination [24]. Nevertheless, a main limitation was the struggle of health staff to maintain the scheduled appointments, especially due to the COVID pandemic, meaning that in various cases, scheduled screening accomplishments were not finalized. Moreover, we used a non-probability convenience sampling, but considering the observational nature of the study, the specific sub-group of population, and the multivariate analysis performed, the risk of bias is minimal. Finally, we cannot know if the vaccinated subjects seronegative at enrollment were primary vaccine failures or they lost circulating antibodies over the years, but in both cases our management should have prevented the disease. Scientific evidence shows that primary vaccine failure for a full cycle of the MMR vaccine is lower that the reported values of serosusceptibility, therefore the mechanism of loss of circulating antibodies over time should be confirmed.

## 5. Conclusions

Low VC among health personnel and the implementation of effective strategies to increase it were reported in many scientific papers [45,46]. Our experience has showed that increasing VC required highly qualified physicians who were experts in vaccinology and occupational medicine, with strong coordination between the Hygiene and the Occupational Medicine departments. Because of this, the sample subjected to vaccination prophylaxis and serological test was small, and the results showed wide 95%Cis. Future studies should repeat these evaluations with a larger sample.

Our protocol, four years after its implementation, showed a high level of performance; indeed, only 13 (2.5%) subjects refused the required vaccine prophylaxis. Moreover, <5% of screened HCWs were still susceptible or did not receive vaccine prophylaxis. Clearly, the principal strength of our protocol was the determination of the suitability for a particular profession subjected to susceptibility status. Subjects who refused immunization did not receive authorization to work in high-risk operative units and therefore, in many cases, they reconsidered their decision in order to become suitable for their desired work. Nevertheless, a small number of subjects eluded vaccination.

The experience reported in our study highlights how a specific vaccination protocol can increase the compliance of healthcare professionals with immunization prophylaxis and make hospital wards safer by reducing the proportion of susceptible HCWs. This evidence confirms that adequate VCs cannot be recorded without the sustenance of specific policies. Indeed, in 2018, the regional government of Apulia approved a law that defined vaccinations for health personnel semi-mandatory, considering work suitability assessed by occupational health doctors, analogous to our procedure implemented in 2017 [47]. Especially during the COVID-19 pandemic, VPDs may still present a hazard in the nosocomial environment. Considering the above-reported experiences, as also claimed in other scientific evidence [27,48], a mandatory strategy might be the most efficient policy in order to reach satisfactory VC among HCWs and therefore keep the nosocomial setting safe.

## Figures and Tables

**Table 1:** Characteristics of the sample.

Variable	Value
Age (mean ± SD; range)	35.7 ± 10.6 (20.0–68.0)
Females; *n* (%)	936 (63.4%)
Chronic disease; *n* (%)	287 (19.4%)
Allergies; *n* (%)	348 (23.6%)
Profession; *n* (%)	
● physician	485 (32.8%)
● nurse	471 (31.9%)
● auxiliary staff	217 (14.7%)
● other	187 (12.7%)
● not reported	117 (7.9%)
Operative Unit; *n* (%)	
● Medical specialty	661 (44.8%)
● Surgery	258 (17.5%)
● Services	306 (20.7%)

**Table 2:** Immunization status of healthcare workers (*n* = 1477) per vaccine type.

Vaccine	*n*	%
Measles		
● No vaccination	994	67.3
● 1 dose	121	8.2
● 2 doses	362	24.5
Mumps		
● No vaccination	1031	69.8
● 1 dose	106	7.2
● 2 doses	340	23.0
Rubella		
● No vaccination	1007	68.2
● 1 dose	125	8.5
● 2 doses	345	23.4
Varicella		
● No vaccination	1425	96.5
● 1 dose	17	1.2
● 2 doses	35	2.3

**Table 3:** Analysis of the determinants of susceptibility to serological evaluation in multivariate logistic regression models.

Determinants	Measles		Mumps		Rubella		Vzv	
	aOR (95%CI)	*p*-Value	aOR (95%CI)	*p*-Value	aOR (95%CI)	*p*-Value	aOR (95%CI)	*p*-Value
Sex (male vs. female)	1.27 (0.89–1.83)	0.193	1.43 (1.01–2.03)	0.049	1.18 (0.80–1.74)	0.394	1.10 (0.69–1.75)	0.697
Age (yrs)	0.91 (0.88–0.94)	<0.0001	0.95 (0.92–0.97)	<0.0001	0.94 (0.92–0.97)	<0.0001	0.98 (0.96–1.01)	0.146
Immunization status								
● 1 dose vs. no vaccination	1.02 (0.57–1.83)	0.955	1.23 (0.69–2.19)	0.476	0.29 (0.13–0.66)	0.003	1.82 (0.39–8.53)	0.449
● 2 doses vs. no vaccination	1.11 (0.72–1.71)	0.629	0.69 (0.43–1.10)	0.119	0.95 (0.06–0.25)	<0.0001	7.48 (3.22–17.36)	<0.0001
Professional category								
● other job vs. physician	0.91 (0.57–1.46)	0.704	1.07 (0.68–1.67)	0.775	1.36 (0.85–2.28)	0.200	1.20 (0.66–2.18)	0.558
● nurse vs. physician	0.63 (0.42–0.95)	0.027	0.88 (0.58–1.32)	0.523	0.95 (0.60–1.53)	0.843	1.27 (0.76–2.14)	0.360
Specialty								
● surgery vs. medical	1.18 (0.71–1.97)	0.529	1.09 (0.67–1.78)	0.719	1.17 (0.68–2.00)	0.571	0.60 (0.28–1.29)	0.192
● service vs. medical	1.29 (0.85–1.97)	0.236	0.93 (0.61–1.42)	0.747	1.12 (0.69–1.82)	0.634	1.40 (0.81–2.42)	0.227
Chronic disease (yes/no)	1.35 (0.89–2.06)	0.161	1.16 (0.75–1.78)	0.505	1.41 (0.89–2.25)	0.144	0.93 (0.53–1.63)	0.795
Allergies (yes/no)	1.34 (0.91–1.96)	0.133	1.21 (0.82–1.78)	0.344	0.75 (0.47–1.19)	0.224	1.21 (0.73–2.00)	0.457
	Chi-square = 6.2; *p* = 0.627		Chi-square = 852.3; *p* = 0.183		Chi-square = 11.3; *p* = 0.184		Chi-square = 5.4; *p* = 0.717	

**Table 4:** Immunization prophylaxis in non-immune HCWs per infection.

Infection	Seronegative HCWs (*n*)	HCWs Offered the Vaccine		HCWs Who Accepted Vaccine Prophylaxis		Re-Titered HCWs		Seroconverted		GMT after Booster(s)
		*n*	%	*n*	%	*n*	%	*n*	%	Mean (95%CI)
Measles	188	153	81.3	150	98.0	61	40.7	54	88.5	73.6 (62.8–118.5)
Mumps	188	124	66.0	120	97.6	52	43.3	43	82.7	38.3 (20.5–71.5)
Rubella	157	97	61.8	95	97.9	38	40.0	36	94.7	59.7 (37.3–97.5)
Varicella	111	82	73.8	77	93.9	29	37.7	25	86.2	636.2 (322.8–1297.2)

**Table 5:** Analysis of determinants of vaccination refusal in a multivariate logistic regression model.

Determinant	aOR	95%CI	*p*-Value
Age (years)	1.16	1.09–1.25	<0.0001
Sex (male vs. female)	0.54	0.12–2.33	0.411
Professional category			
● other job vs. physician	1.21	0.24–6.03	0.820
● nurse vs. physician	2.31	0.47–11.35	0.303
Specialty			
● surgery vs. medical	0.43	0.07–2.68	0.362
● service vs. medical	0.39	0.09–1.75	0.220
Chronic disease (yes/no)	1.24	0.35–4.43	0.737
Allergies (yes/no)	1.26	0.37–4.29	0.711

Chi-square = 5.9; *p* = 0.660.

## Data Availability

Data available on request due to restrictions, e.g., privacy or ethical.
